# Supplementary material for: Mechanisms governing the pioneering and redistribution capabilities of the non-classical pioneer PU.1
Source: Nat Commun. 2020 Jan 21;11:402. doi: 10.1038/s41467-019-13960-2 (PMC6972792; doi:10.1038/s41467-019-13960-2)
Supplement: Supplementary file 7 — Source data [file 41467_2019_13960_MOESM7_ESM.zip › Source_Data/FigureS2/FigureS2B_MotifScanOutput/KmeansCluster3/homerResults/motif2.similar.html]

motif2

## Information for motif2

G
C
T
A
A
G
T
C
A
C
G
T
G
A
C
T
T
C
G
A
A
G
T
C
C
G
A
T
  
Reverse Opposite:  

G
C
T
A
T
C
A
G
A
G
C
T
C
T
G
A
T
G
C
A
C
T
A
G
C
G
A
T
  

|  |  |
| --- | --- |
| p-value: | 1e-611 |
| log p-value: | -1.408e+03 |
| Information Content per bp: | 1.650 |
| Number of Target Sequences with motif | 2838.0 |
| Percentage of Target Sequences with motif | 59.51% |
| Number of Background Sequences with motif | 9158.5 |
| Percentage of Background Sequences with motif | 21.60% |
| Average Position of motif in Targets | 97.7 +/- 57.8bp |
| Average Position of motif in Background | 99.2 +/- 70.9bp |
| Strand Bias (log2 ratio + to - strand density) | -0.0 |
| Multiplicity (# of sites on avg that occur together) | 1.17 |
| Motif File: | file (matrix) reverse opposite |

### Similar de novo motifs found

|  |  |  |  |  |  |  |  |
| --- | --- | --- | --- | --- | --- | --- | --- |
| Rank | Match Score | Redundant Motif | P-value | log P-value | % of Targets | % of Background | Motif file |
| 1 | 0.799 | T C G A T A G C C G A T A G C T T G C A A G T C G A C T C T G A A C G T | 1e-50 | -116.735894 | 5.45% | 1.64% | motif file (matrix) |
| 2 | 0.815 | G C T A A G T C A G C T G C A T C G T A T G A C C G T A T G C A A C G T G T A C | 1e-34 | -79.173044 | 3.27% | 0.90% | motif file (matrix) |
| 3 | 0.828 | C G T A A G T C A C G T A G T C C G T A A G T C A C G T C G T A | 1e-13 | -30.830725 | 2.60% | 1.15% | motif file (matrix) |
